# Large-scale health disparities associated with Lyme disease and human monocytic ehrlichiosis in the United States, 2007–2013

**DOI:** 10.1371/journal.pone.0204609

**Published:** 2018-09-27

**Authors:** Yuri P. Springer, Pieter T. J. Johnson

**Affiliations:** 1 Epidemic Intelligence Service, U.S. Centers for Disease Control and Prevention, Atlanta, Georgia, United States of America; 2 Department of Ecology and Evolutionary Biology, University of Colorado, Boulder, Colorado, United States of America; Kansas State University, UNITED STATES

## Abstract

Promoting health equity is a fundamental public health objective, yet health disparities remain largely overlooked in studies of vectorborne diseases, especially those transmitted by ticks. We sought to identify health disparities associated with Lyme disease and human monocytic ehrlichiosis, two of the most pervasive tickborne diseases within the United States. We used general linear mixed models to measure associations between county-level disease incidence and six variables representing racial/ethnic and socioeconomic characteristics of counties (percent white non-Hispanic; percent with a bachelors degree or higher; percent living below the poverty line; percent unemployed; percent of housing units vacant; per capita number of property crimes). Two ecological variables important to tick demography (percent forest cover; density of white-tailed deer) were included in secondary analyses to contextualize findings. Analyses included data from 2,695 counties in 37 states and the District of Columbia during 2007–2013. Each of the six variables was significantly associated with the incidence of one or both diseases, but the direction and magnitude of associations varied by disease. Results suggested that the incidence of Lyme disease was highest in counties with relatively higher proportions of white and more educated persons and lower poverty and crime rates; the incidence of human monocytic ehrlichiosis was highest in counties with relatively higher proportions of white and less educated persons, higher unemployment rates and lower crime rates. The percentage of housing units vacant was a strong positive predictor for both diseases with a magnitude of association comparable to those between incidence and the ecological variables. Our findings indicate that racial/ethnic and socioeconomic disparities in disease incidence appear to be epidemiologically important features of Lyme disease and human monocytic ehrlichiosis in the United States. Steps to mitigate encroachment of wild flora and fauna into areas with vacant housing might be warranted to reduce disease risk.

## Introduction

Health disparities are differences in disease burdens or health outcomes that occur by gender, race/ethnicity, education, income, disability, geographic location, or sexual orientation [1]. Disparities typically manifest as higher disease burdens or more severe health outcomes experienced by disadvantaged or marginalized groups. Because the magnitude of health disparities generally exceeds that of innate (i.e., biological) health differences among populations, and because they are considered inherently unjust and avoidable, the elimination of health disparities is a fundamental goal of public health practice [2]. Characterizing racial/ethnic and socioeconomic status (SES) health disparities is a critical precursor to identifying their mechanistic underpinnings and designing public health interventions that promote health equity.

In the United States, health disparities have been described for a variety of chronic and infectious conditions [3, 4] but remain largely overlooked in studies of vectorborne diseases. Transmission of many such diseases involves arthropod species (e.g., mosquitoes and ticks) whose biogeography, demography, and behavior are strongly affected by abiotic environmental conditions and the availability of suitable habitats and hosts. As a result, variation in human disease burden is typically explained in terms of these ecological factors [5]. However, a growing number of studies investigating mosquito-borne arboviral diseases in the United States have explicitly considered and documented health disparities. Populations or locations with different racial/ethnic and SES profiles have been shown to vary in mosquito abundance and diversity and in the incidence of arboviral infection in mosquitoes and humans [6–22]. This variation has been attributed to factors including the types and availability of mosquito habitat; knowledge of and motivation to perform mosquito control measures; care seeking behavior; diagnostic, testing, and reporting practices of healthcare providers; and strategies and methods of mosquito abatement employed by vector control agencies. Many of the cited studies report greater mosquito abundance, and higher risk and incidence of arboviral infection, in populations and locations associated with low SES.

Although diseases transmitted by ticks are the most common vectorborne diseases in the United States, limited information is available regarding associated health disparities. A search for published studies of tickborne disease epidemiology in the United States revealed only five that explicitly considered racial/ethnic or SES factors. Three studies focused on human disease burden and identified associations with at least one SES variable (percent of residents living below the poverty line and percent of housing units that were vacant) [23–25]. Two studies focused on the seroprevalence of pathogen antibodies in dogs and one of these found a negative association with median household income of dog owners [26, 27]. The generalizability of these findings is uncertain due to the paucity and narrow spatial extent of associated studies. Of the three that considered human disease burden, all were limited to a small geographic area (a single state or among four contiguous states) representing a fraction of the range of the focal disease in the United States. Additionally, none of the studies compared the epidemiology of multiple tickborne diseases simultaneously.

Here, we describe health disparities associated with two of the most pervasive tickborne diseases in the United States, Lyme disease (LD) and human monocytic ehrlichiosis (HME). Together, LD and HME are estimated to infect over 300,000 persons nationwide annually [28, 29]. Although the etiological agents of these diseases have biologically similar transmission cycles, they are spread by different vector species and are geographically focal in distinct areas. By using county-level counts of cases reported over seven years in all states where the tick vectors of LD and HME are considered established, we assessed the direction and magnitude of associations between disease incidence and six variables describing racial/ethnic and SES characteristics of select United States counties (hereafter, socioeconomic variables). Our goals were to (1) evaluate potential associations between the incidence of LD and HME and each socioeconomic variable, while correcting for county population sizes and spatial autocorrelation, (2) determine whether these associations were consistent or varied by disease, and (3) explore the degree to which observed associations were driven by relationships between incidence and two ecological variables.

## Materials and methods

We obtained the annual number of reported cases of LD and HME in all U.S. counties during 2007–2013 from U.S. Centers for Disease Control and Prevention’s National Notifiable Diseases Surveillance System (S1 Table). This seven-year period was intentionally centered on 2010, the year many of the socioeconomic variables used in our analyses were estimated as part of the decennial U.S. Census. For each disease, we summed the number of reported cases across years within each county (hereafter, case counts) to reduce the influence of temporal variance. Analyses were limited to states in which one or both of the primary tick vectors for LD and HME—*Ixodes scapularis* and *Amblyomma americanum*, respectively—are presumed to be established in at least one county [30, 31]. The associated area includes 2,695 counties in 37 states and the District of Columbia.

The six socioeconomic variables used in our analyses included measures of racial/ethnic composition (percentage of population classified as white, non-Hispanic; hereafter, white), educational achievement (percentage of population aged ≥25 years with a bachelor’s degree or higher; hereafter, education), unemployment (percentage of population [labor force] unemployed; hereafter, unemployment), crime (per capita number of property crimes; hereafter, crime), poverty (percentage of population living below the federal poverty line; hereafter, poverty) and vacant housing (percentage of all housing units that were vacant; hereafter, vacant housing) (S1 Table). These variables were selected either because they are commonly used in area-based studies of health disparities (e.g., education, unemployment, poverty) or because they were included in previous studies of tickborne disease epidemiology in the U.S. that considered socioeconomic factors (e.g., vacant housing [23], crime [32]). Vacant housing is an aggregate measure generated as part of the 2010 decennial U.S. Census that includes four housing vacancy types: for rent; for sale; for seasonal, recreational, or occasional use; for other use. Rather than partitioning the housing variable in our primary and secondary analyses, we included the aggregate measure to facilitate comparison of our findings with those of the other study that considered vacant housing generally [23]. Socioeconomic variables were estimated by using multiple sources for 2010 or a multiyear period centered on 2010. No pairwise correlation coefficients between any of the six socioeconomic variables exceeded *r =* ±0.5.

We also obtained data on two ecological variables related to the tick vectors of LD and HME for use in our analyses (S1 Table). The density of white-tailed deer (*Odocoileus virginianus*; hereafter, deer density) was included because this species is the principal definitive host for both *I*. *scapularis* and *A*. *americanum*. A deer density map [33] was digitized and county-level deer density was estimated based on the percent cover of five density categories (>45/square mile; 30–45/square mile; 15–30/square mile; <15/square mile; rare, absent, or urban area with unknown population). The county-level percent cover of forests (deciduous, evergreen, and mixed forests combined; hereafter, forest cover) [34] was included because this habitat is generally associated with abiotic conditions highly suitable for tick survival and is the preferred habitat of many tick host species. No pairwise correlation coefficients between these two ecological variables and the six socioeconomic variables exceeded *r =* ±0.5.

We modeled case counts of LD and HME using generalized linear mixed models implemented in R software [35] with package lme4 [36]. County population size (from the 2010 decennial U.S. Census) was included as an offset term, effectively changing the response variable from case counts to incidence. Incidence was modeled as an overdispersed Poisson variable for which we included an observation-level random effect to account for expected overdispersion in case counts [37, 38]. We incorporated random intercept terms for state (to account for spatial autocorrelation among counties within the same state because of their proximity or similarities in disease surveillance practices) and region (to account for larger-scale spatial autocorrelation in climate and environmental conditions that might affect tick distribution and abundance). Regions were based on standard federal regions [39], with three pairs of these regions combined (Upper and Lower Northeast, Central Mountain and Upper Midwest, Southcentral and Midwest) because of limited numbers of cases within some of the original regions (S1 Fig).

To evaluate general associations between the socioeconomic variables and the incidence of LD and HME, we first modeled the association between each socioeconomic variable and incidence, specifying disease (LD or HME) as a fixed effect. Observations were thus nested within counties where the diseases were sympatric. Values for each socioeconomic variable were centered by subtracting the mean and scaled by dividing each value by its centered standard deviation. HME was used as the reference category, and we included an interaction term between disease and the socioeconomic variable to determine whether associations differed in direction (positive or negative) or magnitude by disease. Because the interaction term was significant in models for five of six socioeconomic variables (based on a likelihood ratio test, S2 Table), we performed subsequent analyses separately for each disease.

To estimate parameter coefficients, we initially used univariable models and characterized the association between incidence and each socioeconomic variable individually. This functioned to identify and characterize significant associations between patterns of infection and racial/ethnic and SES characteristics of the population in included counties, while accounting for factors such as population size and spatial autocorrelation. All significant terms were subsequently combined into a multivariable mixed model to evaluate the amount of unique variance in infection captured by each; we used sequential backward selection with likelihood ratio tests to identify the best-supported model. After arriving at the final (reduced) multivariable model for each disease, we further ensured a lack of collinearity among predictors by examining variance inflation factors (VIFs) and testing for spatial autocorrelation in the residuals by using spline correlogram and geospatial bubble plots. We treated VIFs >4 as potentially indicative of collinearity. Finally, to assess the degree to which observed associations between incidence and socioeconomic variables were functions of underlying correlations with ecological variables, we added deer density and forest cover (values centered by using the same method applied to socioeconomic variables) to the final multivariable models for each disease, identified the best-supported model as described previously, and examined changes in variable coefficients and significance values between final multivariable models with and without ecology.

We conducted a simplified post hoc analysis to explore the observed associations between disease incidence and vacant housing. For each of the 2,695 counties, we used 2010 decennial U.S. Census data to partition the aggregate vacant housing measure into the percentage of vacant housing associated with each of the four housing vacancy types: for rent (vacant housing units offered for sale or rent, or rented but not occupied); for sale (vacant housing units offered for sale only, or sold but not occupied); for seasonal, recreational, or occasional use (hereafter, seasonal use); for other use (includes all vacant housing not classified as one of the other three types). Data from the U.S. Census Current Population Survey Housing Vacancy Survey (https://www.census.gov/housing/hvs/data/histtab18.xlsx) indicate that the most common characteristics of vacant housing classified as other use in 2012 (data were not available as part of the 2010 decennial U.S. Census) were housing units: kept vacant for personal or family reasons, including for storage (~27%); in need of or being repaired (~23%); in foreclosure, possibly abandoned, or to be demolished/condemned (~18%); being prepared for rental or sale (~7%); associated with extended resident absence (~6%); involved in legal proceedings (~6%). No pairwise correlation coefficients between any of the four housing vacancy type variables exceeded *r =* ±0.2. We modeled case counts of LD and HME using the same generalized linear mixed modeling approach implemented in our primary and secondary analyses. We ran a single model for each disease that included county population size (an offset term), random intercept terms for state and region, and the four housing vacancy type variables (as fixed factors). Associations between disease incidence and the four housing vacancy types were evaluated based on results of the full models (i.e., no sequential backward selection was performed).

## Results

**Fig 1:**
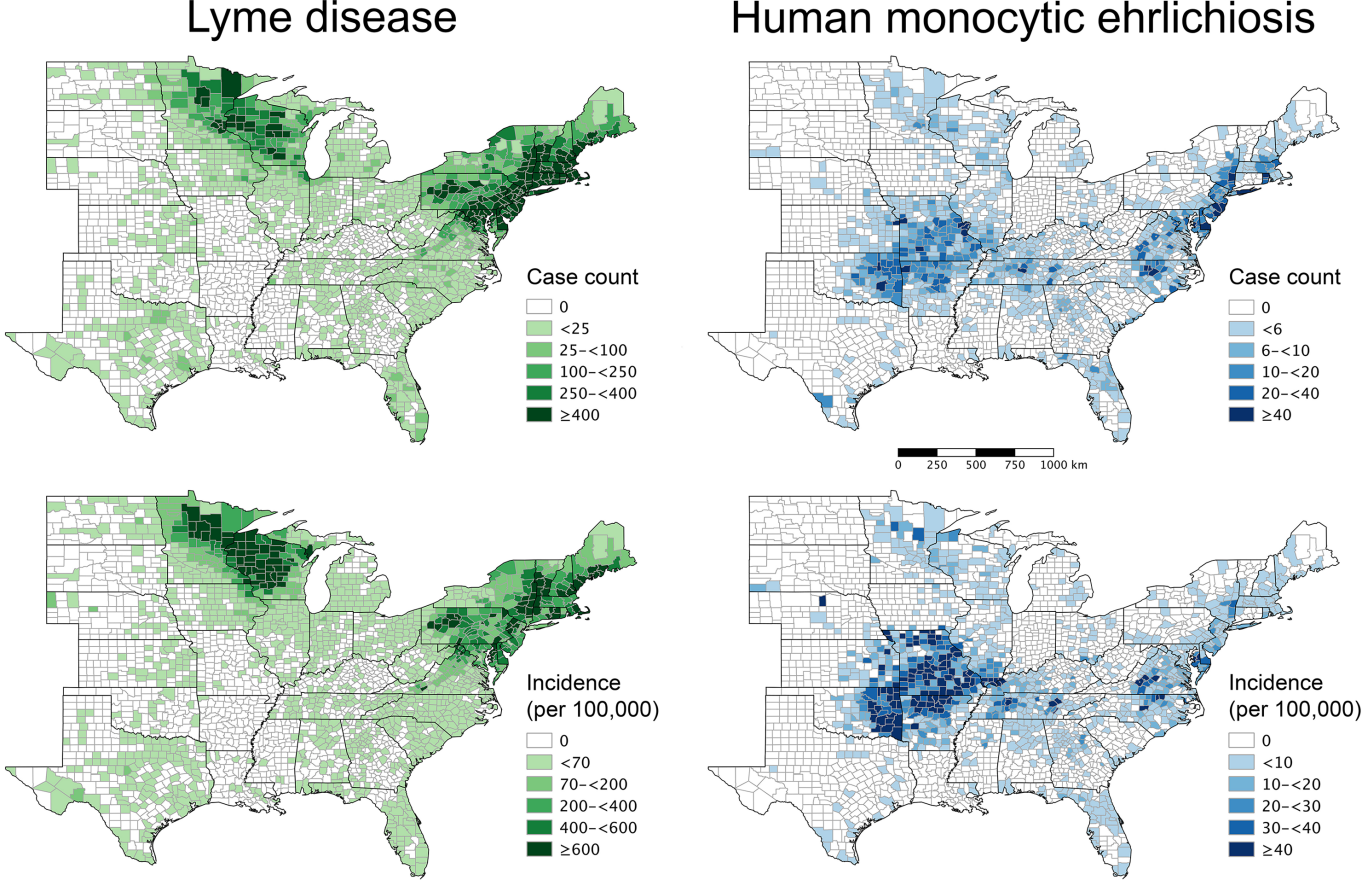
Maps of county-level case counts and incidence of Lyme disease and human monocytic ehrlichiosis in the United States during 2007–2013. Case counts were calculated as the sum of the annual numbers of reported cases of each disease across the seven-year period in each county (N = 2,695 in 37 states including the District of Columbia). Incidence (standardized to cases per 100,000 persons) was calculated as the ratio of case counts and county population size in 2010 (obtained from the 2010 decennial U.S. Census).

During 2007–2013, the median annual numbers of reported cases of LD and HME within all 2,695 counties were 30,796 (range: 26,294–35,870) and 939 (range: 722–1,479), respectively. The total case counts of LD (N = 216,444 cases) and HME (N = 6,865 cases) within these counties represented >99% of all reported cases of each disease throughout the entire United States. The two diseases exhibited distinct geographic foci (Fig 1). For LD, case counts and incidence were highest among counties in the Northeast and upper Midwest; for HME, case counts and incidence were highest among counties in the Midwest/Southcentral and Midatlantic.

**Fig 2:**
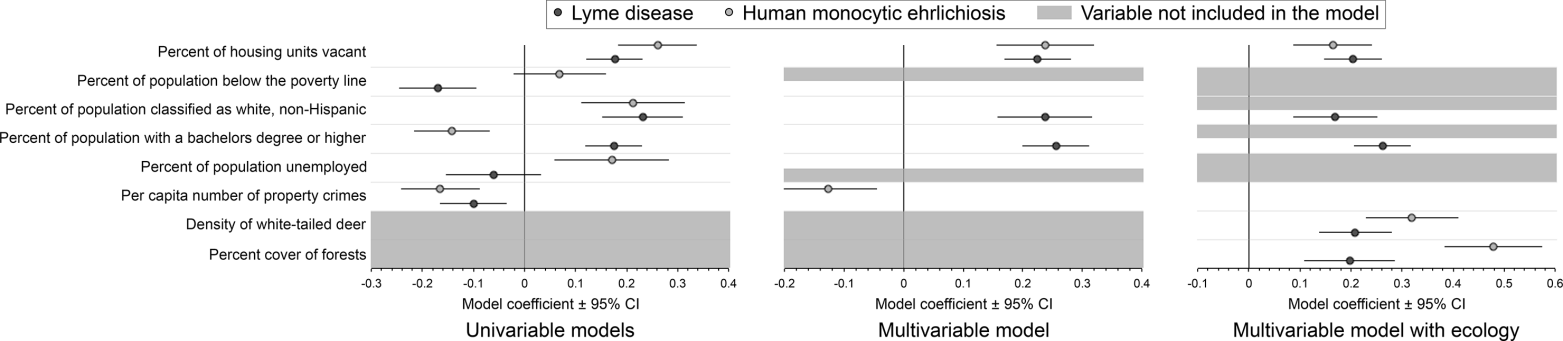
Results of county-level analyses using general linear mixed modeling to quantify associations between the incidence of Lyme disease and human monocytic ehrlichiosis with six racial/ethnic and socioeconomic variables (socioeconomic variables) and two ecological variables. For each disease, results of univariable models (each socioeconomic variable individually) (left), the final (reduced) multivariable model including multiple socioeconomic variables together (middle), and the final (reduced) multivariable model including multiple socioeconomic variables and two ecological variables together (right) are shown. Incidence was modeled using case counts (annual numbers of reported cases of each disease summed during 2007–2013 in each of 2,695 counties in 37 states and the District of Columbia); county population size in 2010 was included in the models as an offset term. Values for socioeconomic and ecological variables were centered by subtracting the mean and scaled by dividing each value by its centered standard deviation. Sources and summary values of disease, socioeconomic and ecological data are provided in S1 Table.

After stratifying the dataset by disease, univariable models for LD identified positive associations between LD incidence and vacant housing, white, and education, and negative associations between LD incidence and both poverty and crime (Fig 2, S3 Table). No significant association between LD incidence and unemployment was identified. In the final multivariable LD model, the positive associations between LD incidence and vacant housing, white, and education were retained. Univariable models for HME identified positive associations between HME incidence and vacant housing, white, and unemployment, and negative associations between HME incidence and both education and crime (Fig 2, S4 Table). No significant association between HME incidence and poverty was identified. In the final multivariable HME model, the positive association between HME incidence and vacant housing, and the negative association between HME incidence and crime, were retained.

When final multivariable models were run with the two ecological variables included, the incidence of both LD and HME was positively correlated with both deer density and forest cover (Fig 2, S3 and S4 Tables). In the final multivariable models with ecology, positive associations between LD incidence and vacant housing, white, and education, and positive associations between HME incidence and vacant housing, were retained; associated coefficients maintained values comparable to those in the models without ecology. Using residuals from the final models, all variance inflation factors were <2, and spatial autotcorrelation was not apparent in spline correlograms or geospatial bubble plots.

**Fig 3:**
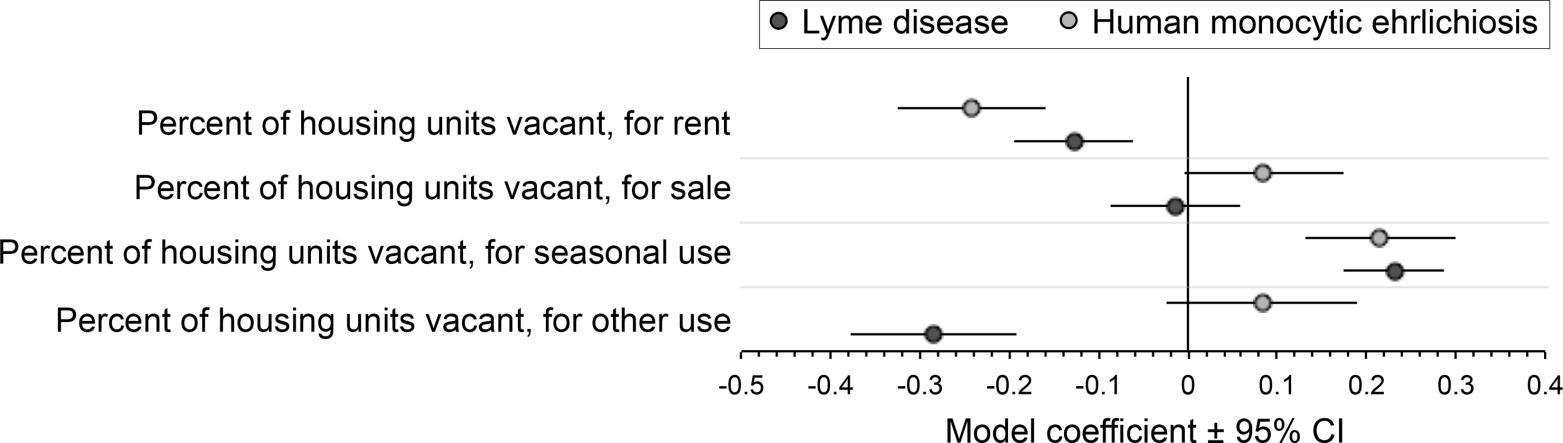
Results of county-level post hoc analyses using general linear mixed modeling to quantify associations between the incidence of Lyme disease and human monocytic ehrlichiosis with four housing vacancy type variables. Results of the non-reduced multivariable models (all four housing vacancy type variables together) are shown. Incidence was modeled using case counts (annual numbers of reported cases of each disease summed during 2007–2013 in each of 2,695 counties in 37 states and the District of Columbia) with county population size in 2010 included in the model as an offset term. Values for housing vacancy type variables were centered by subtracting the mean and scaled by dividing each value by its centered standard deviation. Source and summary values of housing vacancy type data are provided in S1 Table.

In the post hoc analysis involving data on housing vacancy types, the incidence of both LD and HME was positively correlated with the percent of housing units vacant for seasonal use and negatively correlated with the percent of housing units vacant for rent (Fig 3, S5 Table). The incidence of LD was also negatively correlated with the percent of housing units vacant for other use. No significant association between the incidence of either disease and the percent of housing units vacant, for sale was identified. Among the housing vacancy types, housing vacant for other use accounted for the largest percentage on average (35%), followed by housing vacant for seasonal use (28%), for rent (22%), and for sale (15%).

## Discussion

Studies of health disparities are critical to a robust understanding of the patterns and drivers of variation in disease burden and to the development of public health policies that effectively promote health equity. Our analyses identified multiple socioeconomic variables that were associated with the incidence of LD and HME, two of the most pervasive tickborne diseases in the United States. Of the six socioeconomic variables evaluated, which collectively represent a range of racial/ethnic and socioeconomic characteristics of affected populations, all were significantly associated with the incidence of one or both diseases in univariable analyses. Furthermore, the direction or magnitude of some of these associations varied by disease. For example, counties with more educated populations exhibited a significantly higher incidence of LD but a lower incidence of HME. Notably, the magnitude of some associations between incidence and socioeconomic variables was comparable to those involving ecological factors considered fundamental drivers of tickborne disease epidemiology in North America. This was particularly apparent for vacant housing, which was positively associated with the incidence of both LD and HME. Collectively, our findings indicate that racial/ethnic and SES disparities in incidence are geographically widespread and epidemiologically important features of at least two tickborne diseases in the United States.

Two findings of our investigation are particularly noteworthy. First, we identified contrasting health disparities between LD and HME. Models for the two diseases combined indicated variation in either the direction or magnitude of associations with socioeconomic variables by disease. In univaraible analyses, the incidence of both diseases was positively associated with the percentage of county population classified as white, non-Hispanic and negatively associated with the per capita number of property crimes. In addition, HME incidence was positively associated with the percentage of county population that was unemployed and negatively associated with the percentage of county population with a bachelor’s degree or higher. Although the ecological nature of our analyses precludes interpretation at the individual level, these results are consistent with a higher incidence of HME among less-educated whites living in communities with high rates of unemployment and low rates of crime. In contrast, we found evidence of a reverse health disparity for LD; incidence was highest in counties with what would generally be considered to be more socioeconomically advantaged populations. LD incidence was positively associated with the percentage of county population with a bachelor’s degree or higher and negatively associated with the percentage of population living below the poverty line; these results are consistent with a higher incidence of LD among more-educated whites living in relatively affluent communities with low rates of poverty and crime. The mechanisms underpinning these contrasting health disparities between LD and HME are unclear and warrant further investigation.

Second, we found that the incidence of both LD and HME showed a strong positive association with the percentage of housing units in a county that were vacant. The magnitude of these associations was surprisingly large and consistent between diseases. In the final multivariable models with ecology, the magnitude of the vacant housing association was comparable to that of one or both of deer density and forest cover, two ecological factors considered fundamental drivers of the epidemiology of tickborne diseases in North America. The persistent influence of all three variables in multivariable models also suggests that they account for distinctive (non-collinear) variance in case counts. A positive association between vacant housing and incidence was previously documented for HME in Missouri [23]; our findings, produced by analyses involving case counts collected over seven years in 37 states and the District of Columbia, suggest that this association is geographically widespread and consistent in direction and magnitude for at least two tickborne diseases in the United States. Results of our post hoc analysis provide additional insight into the positive associations between disease incidence and vacant housing. The incidence of both diseases was positively associated with the percent of housing units vacant for seasonal use, which accounted for ~30% of all vacant housing on average. No other positive associations were detected between the incidence of either disease and any of the other three housing vacancy type variables. These findings suggest that seasonally vacant homes are likely driving the overall association between disease incidence and vacant housing observed in our primary and secondary analyses.

Multiple mechanisms might underlie the observed associations between disease incidence and vacant housing. In general, ecological recolonization may be occurring in areas with a high proportion of temporarily or permanently vacant structures [40]. When this process of greening involves the (re)establishment of vector populations in and around homes, either directly or via the presence of nonhuman hosts (e.g., rodents and other small mammals for many tick species), increases in rates of infection among humans can result. For example, Reisen et al. [9] reported concomitant increases in rates of mortgage delinquency and home abandonment driven by the U.S. subprime mortgage crisis, the number of abandoned swimming pools, abundance of *Culex tarsalis* in impacted urban neighborhoods, and the incidence of human cases of West Nile virus in Kern county, California in 2007. The extent of greening will depend on the timing, frequency, and duration of vacancies as well as on the management of associated structures and adjacent areas (e.g., landscaping, pest control). In addition to the physical condition of vacant houses and their surrounding environments, observed associations are almost certainly driven in part by the behavior of residents when they are present. In particular, occupants of seasonally used vacation homes likely spend a relatively large portion of time recreating outdoors, increasing their probability of encountering ticks; exposure risk could be further amplified by the aforementioned effects of peridomestic greening.

This investigation constitutes the first large-scale comparative evaluation of racial/ethnic and SES factors associated with disparities in the incidence of two tickborne diseases in North America. Our findings reinforce and extend those of the few previous studies of tickborne diseases in the United States that considered health disparities, albeit generally within geographically narrower frameworks. In a county-level study of HME in Missouri, Bayles and Allan [23] found positive associations between incidence and vacant housing, race (proportion white), poverty (proportion living below the poverty line), occupation (proportion working in outdoor occupations such as agriculture and forestry), and in contrast to our results, education (proportion with high-school-level diploma). Raghavan and colleagues found positive associations between county-level poverty (proportion living below the poverty line) and incidence, but failed to detect significant associations between incidence and mean housing age (year structure was built), in analyses of HME in Kansas [25] and of Rocky Mountain spotted fever in four contiguous states (Kansas, Missouri, Oklahoma, and Arkansas) [24]. In contrast to the paucity of studies of these types in the United States, a number of investigations of tickborne encephalitis (TBE) in Europe have included SES variables in epidemiologic analyses. Increases in TBE incidence occurred in many areas of eastern and central Europe following the collapse of the Soviet Union, and low SES has been identified as an important risk factor for infection [41–43]. Acarological risk is highest in forest environments where ticks are abundant, and the end of communist rule appears to have precipitated a surge in the percentage of persons collecting forest-derived food resources for commercial and subsistence purposes. Forest visitation frequency has been positively correlated with unemployment and is highest among persons with lower income and less education [44–46]. These persons are also less likely to be vaccinated against TBE [46, 47]. While these studies are relatively few in number, their consistent detection of associations between the risk of exposure to or incidence of tickborne diseases and SES characteristics suggests that health disparities are features of these systems that should be considered with greater frequency in epidemiologic investigations.

Interpretation of our findings should be made within a framework defined by the scale and nature of our analyses. We evaluated associations on average across particular counties and years, and documented patterns could be different in other locations and at other times. While our results may be consistent with racial/ethnic and SES characteristics of persons living in associated counties during the time period examined, actual characteristics of cases included in our analyses were not considered. Documented associations are therefore specific to counties and their aggregate populations and cannot be extrapolated to the level of individual persons. Although our use of county-level data might have masked within-county heterogeneity in socioeconomic and ecological characteristics in some instances, case count data were not available at a spatial scale below the county level. One potential limitation of our investigation is the fact that case count data might be biased by spatiotemporal variation in care seeking behavior or medical diagnostic and testing practices. For example, in areas where a disease is rare, relatively low awareness of or familiarity with signs and symptoms of infection might lead to lower frequencies of cases seeking care and clinicians correctly diagnosing or requesting confirmatory testing for infections. Both of these phenomena would result in underestimates of disease burden. The reverse might be true in areas where a disease is common. To help offset the effects of this limitation, we based our analyses on case counts collected over seven years (reducing zero-inflation problems), liberally estimated vector distributions to include a large number of counties and geographic areas, and incorporated random intercept terms for state and region in our models. As a result of this approach, our analyses did include counties in which one or both tick vector species are not known to occur. To ensure that these counties did not bias our results and lead to the identification of spurious associations, we reran our disease specific models using only the subset of counties in which the associated tick vector species was classified as established or reported [30, 31]. Results of these subset analyses (S2 Fig, S6 and S7 Tables) were essentially identical to those of the full analyses involving all 2,695 counties in terms of the identity, magnitude, and direction of documented associations. The handful of minor differences between analyses suggested that the full analyses likely masked some disparities and were thus relatively conservative. For example, in the final multivariable models produced by the subset analyses, a positive association with white was retained for HME and a negative association with crime was retained for LD, whereas neither of these associations were apparent in the final multivariable models generated by the full analyses.

Our results elucidate variation in the incidence of tickborne diseases from a rarely considered perspective and inform efforts to mitigate disparities in, and the absolute burden of, tickborne diseases in the United States. Specifically, investigation findings are consistent with at least three potential recommendations for the management and study of tickborne diseases in the United States. First, residents and public health practitioners should be mindful of possible associations between vacant housing and tickborne disease risk. In some instances, steps to prevent or reduce the encroachment of wild flora and fauna into areas with vacant housing might be warranted to slow greening and reduce disease risk. Second, public education campaigns intended to reduce acarological risk (e.g., identifying habitats and times of day or year associated with higher probabilities of tick exposure, clarifying when and how to perform tick checks) may be especially valuable in communities with a high percentage of vacant housing in general or of seasonally vacant housing in particular. More broadly, public health practitioners should be cognizant of variation in the SES profiles of high incidence communities when conducting such educational outreach as receptivity to public health recommendations can vary between high and low SES populations [14]. Associated messaging can be tailored to the education levels, activity patterns, priorities, and socioeconomic constraints of affected populations. Finally, studies of tickborne diseases at finer geographic scales (e.g., within counties) are needed to verify and elucidate the mechanisms underpinning the health disparities reported herein. The small number of cases at these scales will limit the statistical power of correlative studies like ours. Instead, hypothesis-driven observational studies or experimental manipulations could provide more focused and powerful mechanistic evaluations of health disparities associated with tickborne diseases. Results of such investigations would more appropriately and effectively inform targeted public health action to reduce infection risk and associated disparities at the local level.

## Supporting information

S1 Fig Map of the boundaries of geographic regions used in the analyses and based on the standard federal regions established by the United States Office of Management and Budget. Upper Northeast and Lower Northeast standard federal regions (regions I and II, respectively) are combined into the Northeast geographic region; Central Mountain and Upper Midwest standard federal regions (regions VIII and V, respectively) are combined into the Upper Midwest geographic region; Southcentral and Midwest standard federal regions (regions VI and VII, respectively) are combined into the Midwest/Southcentral geographic region; Midatlantic and Southeast regions correspond to standard federal regions III and IV, respectively.
(TIF)

S2 Fig Results of county-level subset analyses using general linear mixed modeling to quantify associations between the incidence of Lyme disease (LD) and human monocytic ehrlichiosis (HME) with six racial/ethnic and socioeconomic variables (socioeconomic variables) and two ecological variables. For each disease, subset analyses were limited to the subset of the 2,695 counties included in the full analyses in which the primary tick vector for the disease—*Ixodes scapularis* for LD, *Amblyomma americanum* for HME—is presumed to be established or has been reported [30, 31]. The associated area includes 1,421 counties for LD (N = 843 counties with established status, N = 578 counties with reported status) and 1,295 counties for HME (N = 651 counties with established status, N = 644 counties with reported status). For each disease, results of univariable models (each socioeconomic variable individually) (left), the final (reduced) multivariable model including multiple socioeconomic variables together (middle), and the final (reduced) multivariable model including multiple socioeconomic variables and two ecological variables together (right) are shown. Incidence was modeled using case counts (annual numbers of reported cases of each disease summed during 2007–2013 in each of the counties described above); county population size in 2010 was included in the models as an offset term. Values for socioeconomic and ecological variables were centered by subtracting the mean and scaled by dividing each value by its centered standard deviation. Sources of disease, socioeconomic and ecological data are provided in S1 Table.
(TIF)

S1 Table Summary information for variables included in the analyses. Qualitative summary information includes variable type, name, definition and data source(s). Quantitative summary information includes the county-level mean (±95% confidence interval), median, and range of values for each variable among the 2,695 counties included in primary and secondary analyses.
(PDF)

S2 Table Results of county-level analyses using general linear mixed modeling to quantify associations between disease (Lyme disease, human monocytic ehrlichiosis), six racial/ethnic and socioeconomic variables (socioeconomic variables), and their interaction in predicting disease incidence. HME was used as the reference category. Incidence was modeled using case counts (annual numbers of reported cases of each disease summed during 2007–2013 in each of 2,695 counties in 37 states and the District of Columbia); county population size in 2010 was included in the models as an offset term. Values for socioeconomic and ecological variables were centered by subtracting the mean and scaled by dividing each value by its centered standard deviation. Significance was evaluated using likelihood ratio tests. Sources and summary values of disease and socioeconomic data are provided in S1 Table.
(PDF)

S3 Table Results of county-level analyses using general linear mixed modeling to quantify associations between the incidence of Lyme disease with six racial/ethnic and socioeconomic variables (socioeconomic variables) and two ecological variables. Results of univariable models (each socioeconomic variable individually), the final (reduced) multivariable model including multiple socioeconomic variables together, and the final (reduced) multivariable model including multiple socioeconomic variables and two ecological variables together are provided. Incidence was modeled using case counts (annual numbers of reported cases of Lyme disease summed during 2007–2013 in each of 2,695 counties in 37 states and the District of Columbia); county population size in 2010 was included in the models as an offset term. Values for socioeconomic and ecological variables were centered by subtracting the mean and scaled by dividing each value by its centered standard deviation. Sources and summary values of all variables are provided in S1 Table.
(PDF)

S4 Table Results of county-level analyses using general linear mixed modeling to quantify associations between the incidence of human monocytic ehrlichiosis with six racial/ethnic and socioeconomic variables (socioeconomic variables) and two ecological variables. Results of univariable models (each socioeconomic variable individually), the final (reduced) multivariable model including multiple socioeconomic variables together, and the final (reduced) multivariable model including multiple socioeconomic variables and two ecological variables together are provided. Incidence was modeled using case counts (annual numbers of reported cases of human monocytic ehrlichiosis summed during 2007–2013 in each of 2,695 counties in 37 states and the District of Columbia); county population size in 2010 was included in the models as an offset term. Values for socioeconomic and ecological variables were centered by subtracting the mean and scaled by dividing each value by its centered standard deviation. Sources and summary values of all variables are provided in S1 Table.
(PDF)

S5 Table Results of county-level post hoc analyses using general linear mixed modeling to quantify associations between the incidence of Lyme disease and human monocytic ehrlichiosis with four housing vacancy type variables. For each disease, results of the non-reduced multivariable model including all four housing vacancy type variables together are shown. Incidence was modeled using case counts (annual numbers of reported cases of each disease summed during 2007–2013 in each of 2,695 counties in 37 states and the District of Columbia); county population size in 2010 was included in the models as an offset term. Values for housing vacancy type variables were centered by subtracting the mean and scaled by dividing each value by its centered standard deviation. Source and summary values of disease and housing vacancy type data are provided in S1 Table.
(PDF)

S6 Table Results of county-level subset analyses using general linear mixed modeling to quantify associations between the incidence of Lyme disease with six racial/ethnic and socioeconomic variables (socioeconomic variables) and two ecological variables. **Subset analyses were limited to the subset of the 2,695 counties included in the full analyses in which *Ixodes scapularis*, the primary tick vector for Lyme disease, is presumed to be established or reported [30].** The associated area includes 1,421 counties (N = 843 counties with established status, N = 578 counties with reported status). Results of univariable models (each socioeconomic variable individually), the final (reduced) multivariable model including multiple socioeconomic variables together, and the final (reduced) multivariable model including multiple socioeconomic variables and two ecological variables together are provided. Incidence was modeled using case counts (annual numbers of reported cases of Lyme disease summed during 2007–2013 in each county); county population size in 2010 was included in the models as an offset term. Values for socioeconomic and ecological variables were centered by subtracting the mean and scaled by dividing each value by its centered standard deviation. Sources of all variables are provided in S1 Table.
(PDF)

S7 Table Results of county-level subset analyses using general linear mixed modeling to quantify associations between the incidence of human monocytic ehrlichiosis with six racial/ethnic and socioeconomic variables (socioeconomic variables) and two ecological variables. **Subset analyses were limited to the subset of the 2,695 counties included in the full analyses in which *Amblyomma americanum*, the primary tick vector for human monocytic ehrlichiosis, is presumed to be established or reported [31].** The associated area includes 1,295 counties (N = 651 counties with established status, N = 644 counties with reported status). Results of univariable models (each socioeconomic variable individually), the final (reduced) multivariable model including multiple socioeconomic variables together, and the final (reduced) multivariable model including multiple socioeconomic variables and two ecological variables together are provided. Incidence was modeled using case counts (annual numbers of reported cases of human monocytic ehrlichiosis summed during 2007–2013 in each county); county population size in 2010 was included in the models as an offset term. Values for socioeconomic and ecological variables were centered by subtracting the mean and scaled by dividing each value by its centered standard deviation. Sources of all variables are provided in S1 Table.
(PDF)

S1 File Microsoft Excel file containing all raw data used in the analyses. (XLSX)
