# Supplementary figures and images for: Large-scale health disparities associated with Lyme disease and human monocytic ehrlichiosis in the United States, 2007–2013
Source: PLoS One. 2018 Sep 27;13(9):e0204609. doi: 10.1371/journal.pone.0204609 (PMC6160131; doi:10.1371/journal.pone.0204609)

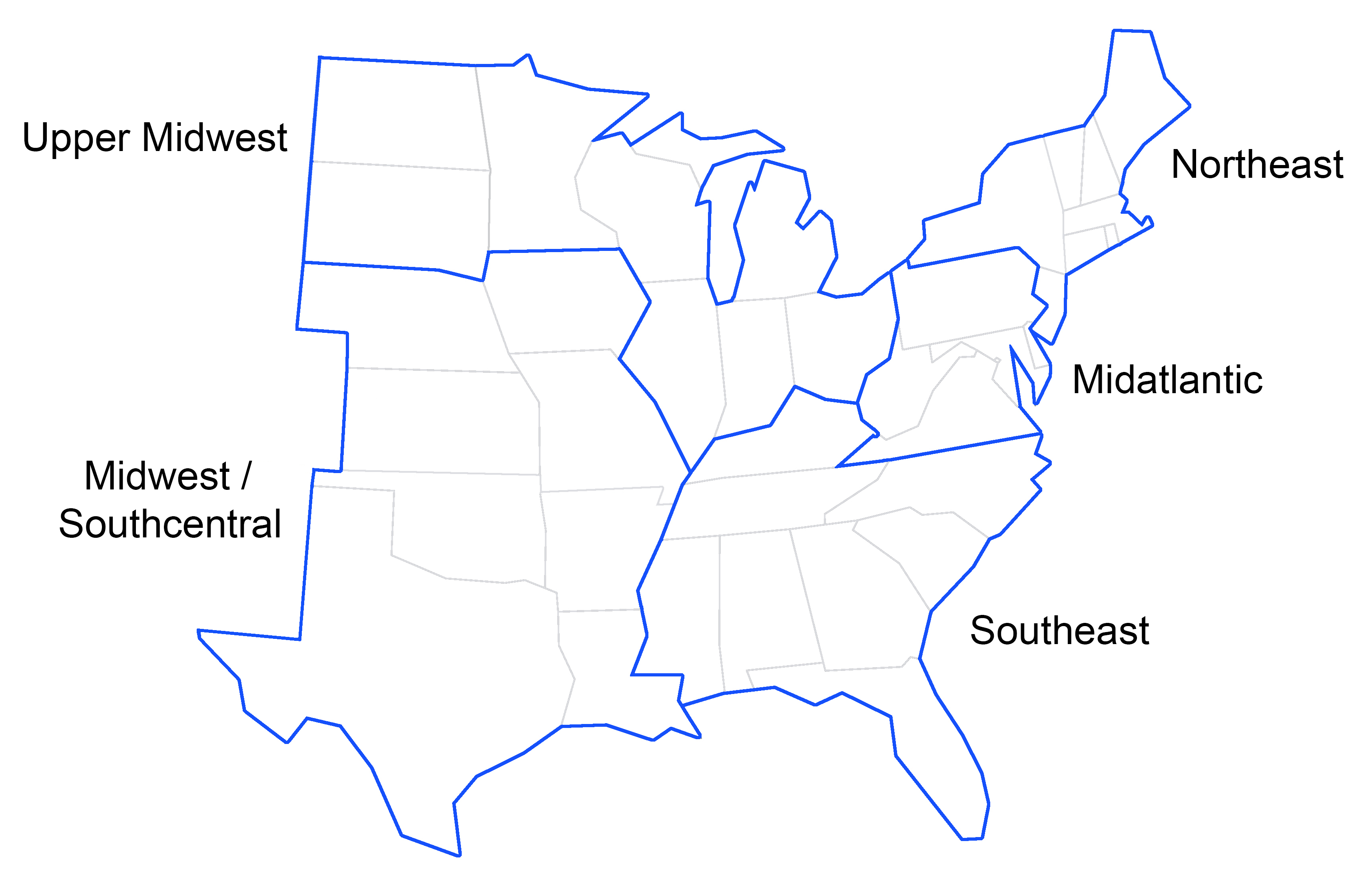

Supplement: S1 Fig — Upper Northeast and Lower Northeast standard federal regions (regions I and II, respectively) are combined into the Northeast geographic region; Central Mountain and Upper Midwest standard federal regions (regions VIII and V, respectively) are combined into the Upper Midwest geographic region; Southcentral and Midwest standard federal regions (regions VI and VII, respectively) are combined into the Midwest/Southcentral geographic region; Midatlantic and Southeast regions correspond to standard federal regions III and IV, respectively. (TIF) [file pone.0204609.s001.tif]

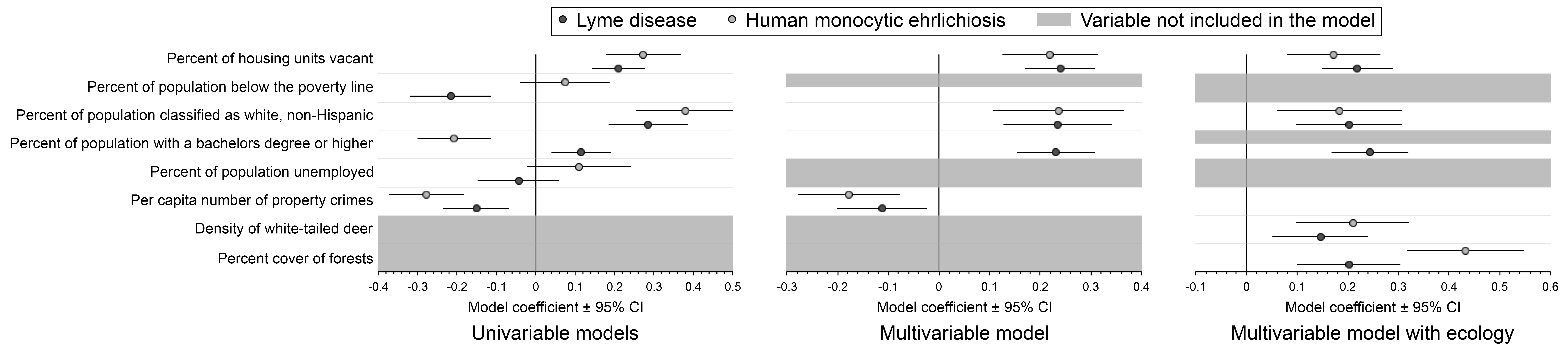

Supplement: S2 Fig — For each disease, subset analyses were limited to the subset of the 2,695 counties included in the full analyses in which the primary tick vector for the disease—Ixodes scapularis for LD, Amblyomma americanum for HME—is presumed to be established or has been reported [30, 31]. The associated area includes 1,421 counties for LD (N = 843 counties with established status, N = 578 counties with reported status) and 1,295 counties for HME (N = 651 counties with established status, N = 644 counties with reported status). For each disease, results of univariable models (each socioeconomic variable individually) (left), the final (reduced) multivariable model including multiple socioeconomic variables together (middle), and the final (reduced) multivariable model including multiple socioeconomic variables and two ecological variables together (right) are shown. Incidence was modeled using case counts (annual numbers of reported cases of each disease summed during 2007–2013 in each of the counties described above); county population size in 2010 was included in the models as an offset term. Values for socioeconomic and ecological variables were centered by subtracting the mean and scaled by dividing each value by its centered standard deviation. Sources of disease, socioeconomic and ecological data are provided in S1 Table. (TIF) [file pone.0204609.s002.tif]
