# Supplementary material for: Large-scale health disparities associated with Lyme disease and human monocytic ehrlichiosis in the United States, 2007–2013
Source: PLoS One. 2018 Sep 27;13(9):e0204609. doi: 10.1371/journal.pone.0204609 (PMC6160131; doi:10.1371/journal.pone.0204609)
Supplement: S1 Table — Qualitative summary information includes variable type, name, definition and data source(s). Quantitative summary information includes the county-level mean (±95% confidence interval), median, and range of values for each variable among the 2,695 counties included in primary and secondary analyses. (PDF) [file pone.0204609.s003.pdf]

S1 Table. Summary information for variables included in the analyses.

Variable type	Variable name	Definition	Data source	Mean (±95% CI)	Median	Range
Response	Disease	For each disease (Lyme disease [LD], human monocytic ehrlichiosis [HME]), sum of annual counts of reported cases during 2007–2013.	U.S. Centers for Disease Control and Prevention (CDC), National Notifiable Diseases Surveillance System. Data were obtained by request from CDC's bacterial diseases and rickettsial zoonoses branches.	LD: 80.3±13.1<br>HME: 2.55±0.30	1<br>0	0–4,865<br>0–168
Socioeconomic	Vacant housing	Percentage of housing units that were vacant in 2010.	U.S. Census Bureau, 2010 decennial U.S. Census.	15.8%±0.35%	13.0%	4.0%–74.0%
Socioeconomic	Poverty	Percentage of the population living below the federal poverty line during 2008-2012.	U.S. Census Bureau, 2012 American Community Survey (5-year estimate, 2008-2012).	39.7%±0.30%	40.0%	0.0%–70.0%
Socioeconomic	White	Percentage of the population classified as white, non-Hispanic in 2010.	U.S. Census Bureau, 2010 decennial U.S. Census.	79.2%±0.73%	87.0%	3.0%–99.0%
Socioeconomic	Education	Percentage of the population aged ≥25 years with a bachelor's degree or higher during 2008-2012.	U.S. Census Bureau, 2012 American Community Survey (5-year estimate, 2008-2012).	19.0%±0.32%	17.0%	4.0%–73.0%
Socioeconomic	Unemployment	Percentage of the population [labor force] that was unemployed in 2010.	U.S. Bureau of Labor Statistics, 2010 local area unemployment statistics.	9.4%±0.12%	9.0%	2.0%–26.0%

Variable type	Variable name	Definition	Data source	Mean (+95% CI)	Median	Range
Socioeconomic	Crime	Per capita number of property crimes reported in 2010.	Counts of crimes were obtained from the U.S. Federal Bureau of Investigations, 2010 uniform crime reporting statistics. County population size[†] data used to calculate per capita number of crimes were obtained from the U.S. Census Bureau, 2010 decennial U.S. Census.	Crimes: 2553.1+322.7<br>County population: 87,866.4+8,804.8	511<br>26,203	0–189,851<br>82–5,194,675
Ecological	Deer density	Density of white-tailed deer *(Odocoileus virginianus)* per square mile in 2008.	Quality Deer Management Association, 2008 white-tailed deer density map (digitized).	2.92+0.04	3.0	1.0–5.0
Ecological	Forest cover	Percent cover of deciduous, evergreen, and mixed forests (combined) in 2011.	Multi-Resolution Land Characteristics Consortium, 2011 National Land Cover Database.	30.3%+0.01%	26.0%	0.0%–93.0%
Housing vacancy type	For rent	Percent of housing units vacant, for rent.	U.S. Census Bureau, 2010 decennial U.S. Census.	22.3%+0.46%	20.7%	0.0%–76.7%
Housing vacancy type	For sale	Percent of housing units vacant, for sale.	U.S. Census Bureau, 2010 decennial U.S. Census.	14.5%+0.27%	13.9%	0.0%–46.6%
Housing vacancy type	For seasonal use	Percent of housing units vacant, for seasonal, recreational, or occasional use.	U.S. Census Bureau, 2010 decennial U.S. Census.	28.2%+0.85%	20.3%	0.0%–96.1%
Housing vacancy type	For other use	Percent of housing units vacant, for other use.	U.S. Census Bureau, 2010 decennial U.S. Census.	35.0%+0.55%	35.5%	0.015%–95.8%

[†] County population size data were included in models as an offset term, changing the response variable from case counts to incidence.
