# Supplementary material for: Large-scale health disparities associated with Lyme disease and human monocytic ehrlichiosis in the United States, 2007–2013
Source: PLoS One. 2018 Sep 27;13(9):e0204609. doi: 10.1371/journal.pone.0204609 (PMC6160131; doi:10.1371/journal.pone.0204609)
Supplement: S2 Table — HME was used as the reference category. Incidence was modeled using case counts (annual numbers of reported cases of each disease summed during 2007–2013 in each of 2,695 counties in 37 states and the District of Columbia); county population size in 2010 was included in the models as an offset term. Values for socioeconomic and ecological variables were centered by subtracting the mean and scaled by dividing each value by its centered standard deviation. Significance was evaluated using likelihood ratio tests. Sources and summary values of disease and socioeconomic data are provided in S1 Table. (PDF) [file pone.0204609.s004.pdf]

S2 Table. Results of county-level analyses using general linear mixed modeling to quantify associations between disease (Lyme disease, human monocytic ehrlichiosis), six racial/ethnic and socioeconomic variables (socioeconomic variables), and their interaction in predicting disease incidence.

	Test of interaction terms	
**Variable**	**Coefficient, SE**	**P value**
Disease	1.8071, 0.0675	<0.0001
Percent of housing units vacant	0.1307, 0.0515	0.0111
Percent of housing units vacant X Disease	0.0955, 0.0650	0.1423
Disease	1.6665, 0.0656	<0.0001
Percent of population living below the poverty line	0.6150, 0.0546	<0.0001
Percent of population living below the poverty line X Disease	-1.2761, 0.0700	<0.0001
Disease	1.7937, 0.0677	<0.0001
Percent of population classified as white, non-Hispanic	0.0845, 0.0622	0.1742
Percent of population classified as white, non-Hispanic X Disease	0.2238, 0.0724	0.0020
Disease	1.6094, 0.0661	<0.0001
Percent of population with a bachelors degree or higher	-0.5011, 0.0465	<0.0001
Percent of population with a bachelors degree or higher X Disease	0.9933, 0.0588	<0.0001
Disease	1.8524, 0.0680	<0.0001
Percent of population unemployed	0.4198, 0.0683	<0.0001
Percent of population unemployed X Disease	-0.6408, 0.0775	<0.0001
Disease	1.8419, 0.0689	<0.0001
Per capita number of property crimes	0.0357, 0.0536	0.5057
Per capita number of property crimes X Disease	-0.2063, 0.0684	0.0026
