# Supplementary material for: Large-scale health disparities associated with Lyme disease and human monocytic ehrlichiosis in the United States, 2007–2013
Source: PLoS One. 2018 Sep 27;13(9):e0204609. doi: 10.1371/journal.pone.0204609 (PMC6160131; doi:10.1371/journal.pone.0204609)
Supplement: S3 Table — Results of univariable models (each socioeconomic variable individually), the final (reduced) multivariable model including multiple socioeconomic variables together, and the final (reduced) multivariable model including multiple socioeconomic variables and two ecological variables together are provided. Incidence was modeled using case counts (annual numbers of reported cases of Lyme disease summed during 2007–2013 in each of 2,695 counties in 37 states and the District of Columbia); county population size in 2010 was included in the models as an offset term. Values for socioeconomic and ecological variables were centered by subtracting the mean and scaled by dividing each value by its centered standard deviation. Sources and summary values of all variables are provided in S1 Table. (PDF) [file pone.0204609.s005.pdf]

S3 Table. Results of county-level analyses using general linear mixed modeling to quantify associations between the incidence of Lyme disease with six racial/ethnic and socioeconomic variables (socioeconomic variables) and two ecological variables.

	Univariable models			Multivariable model		Multivariable model with ecology	
Variable	Coefficient, SE	P value	R^2 value of model	Coefficient, SE	P value	Coefficient, SE	P value
Percent of housing units vacant	0.1766, 0.0279	<0.0001	0.8379	0.2246, 0.0287	<0.0001	0.2040, 0.0291	<0.0001
Percent of population living below the poverty line	-0.1701, 0.0386	<0.0001	0.8293				
Percent of population classified as white non-Hispanic	0.2318, 0.0404	<0.0001	0.8372	0.2371, 0.0405	<0.0001	0.1694, 0.0420	<0.0001
Percent of population with a bachelors degree or higher	0.1749, 0.0286	<0.0001	0.8269	0.2557, 0.0287	<0.0001	0.2622, 0.0283	<0.0001
Percent of population unemployed	-0.0614, 0.0478	0.1990	0.8343				
Per capita number of property crimes	-0.0999, 0.0333	0.0027	0.8362				
Density of white-tailed deer						0.2087, 0.0365	<0.0001
Percent cover of forests						0.1978, 0.0452	<0.0001
**Conditional estimated R^2 value of model**				0.8302		0.8364	

(light grey)	Not included in model
(dark grey)	Included but not significant in final (reduced) model
