# Supplementary material for: Large-scale health disparities associated with Lyme disease and human monocytic ehrlichiosis in the United States, 2007–2013
Source: PLoS One. 2018 Sep 27;13(9):e0204609. doi: 10.1371/journal.pone.0204609 (PMC6160131; doi:10.1371/journal.pone.0204609)
Supplement: S5 Table — For each disease, results of the non-reduced multivariable model including all four housing vacancy type variables together are shown. Incidence was modeled using case counts (annual numbers of reported cases of each disease summed during 2007–2013 in each of 2,695 counties in 37 states and the District of Columbia); county population size in 2010 was included in the models as an offset term. Values for housing vacancy type variables were centered by subtracting the mean and scaled by dividing each value by its centered standard deviation. Source and summary values of disease and housing vacancy type data are provided in S1 Table. (PDF) [file pone.0204609.s007.pdf]

S5 Table. Results of county-level post hoc analyses using general linear mixed modeling to quantify associations between the incidence of Lyme disease and human monocytic ehrlichiosis with four housing vacancy type variables.

	Lyme disease		Human monocytic ehrlichiosis	
**Variable**	**Coefficient, SE**	**P value**	**Coefficient, SE**	**P value**
Percent of housing units vacant, for rent	-0.1275, 0.0340	<0.0001	-0.2423, 0.0425	<0.0001
Percent of housing units vacant, for sale	-0.0143, 0.0371	0.6993	0.0847, 0.0457	0.6400
Percent of housing units vacant, for seasonal use	0.2307, 0.0285	<0.0001	0.2151, 0.0432	<0.0001
Percent of housing units vacant, for other use	-0.2839, 0.0473	<0.0001	0.0837, 0.0544	<0.0001
