# Supplementary material for: Large-scale health disparities associated with Lyme disease and human monocytic ehrlichiosis in the United States, 2007–2013
Source: PLoS One. 2018 Sep 27;13(9):e0204609. doi: 10.1371/journal.pone.0204609 (PMC6160131; doi:10.1371/journal.pone.0204609)
Supplement: S6 Table — Subset analyses were limited to the subset of the 2,695 counties included in the full analyses in which Ixodes scapularis, the primary tick vector for Lyme disease, is presumed to be established or reported [30]. The associated area includes 1,421 counties (N = 843 counties with established status, N = 578 counties with reported status). Results of univariable models (each socioeconomic variable individually), the final (reduced) multivariable model including multiple socioeconomic variables together, and the final (reduced) multivariable model including multiple socioeconomic variables and two ecological variables together are provided. Incidence was modeled using case counts (annual numbers of reported cases of Lyme disease summed during 2007–2013 in each county); county population size in 2010 was included in the models as an offset term. Values for socioeconomic and ecological variables were centered by subtracting the mean and scaled by dividing each value by its centered standard deviation. Sources of all variables are provided in S1 Table. (PDF) [file pone.0204609.s008.pdf]

S6 Table. Results of county-level subset analyses using general linear mixed modeling to quantify associations between the incidence of Lyme disease with six racial/ethnic and socioeconomic variables (socioeconomic variables) and two ecological variables. Subset analyses were limited to the subset of the 2,695 counties included in the full analyses in which *Ixodes scapularis,* the primary tick vector for Lyme disease, is presumed to be established or reported [30].

	Univariable models			Multivariable model		Multivariable model with ecology	
**Variable**	**Coefficient, SE**	**P value**	**R^2 value of model**	**Coefficient, SE**	**P value**	**Coefficient, SE**	**P value**
Percent of housing units vacant	0.2095, 0.0345	<0.0001	0.8327	0.2399, 0.0352	<0.0001	0.2192, 0.0359	<0.0001
Percent of population living below the poverty line	-0.2168, 0.0525	<0.0001	0.8441				
Percent of population classified as white non-Hispanic	0.2848, 0.0516	<0.0001	0.8429	0.2346, 0.0548	<0.0001	0.2026, 0.0536	0.0002
Percent of population with a bachelors degree or higher	0.1151, 0.0389	0.0031	0.8359	0.2310, 0.0390	<0.0001	0.2443, 0.0387	<0.0001
Percent of population unemployed	-0.0432, 0.0529	0.4140	0.8396				
Per capita number of property crimes	-0.1517, 0.0429	0.0004	0.8416	-0.1125, 0.0452	0.0127		
Density of white-tailed deer						0.1461, 0.0481	0.0024
Percent cover of forests						0.2022, 0.0519	<0.0001
**Conditional estimated R^2 value of model**				0.8388		0.8451	

Not included in model

Included but not significant in final (reduced) model
