# Supplementary material for: Large-scale health disparities associated with Lyme disease and human monocytic ehrlichiosis in the United States, 2007–2013
Source: PLoS One. 2018 Sep 27;13(9):e0204609. doi: 10.1371/journal.pone.0204609 (PMC6160131; doi:10.1371/journal.pone.0204609)
Supplement: S7 Table — Subset analyses were limited to the subset of the 2,695 counties included in the full analyses in which Amblyomma americanum, the primary tick vector for human monocytic ehrlichiosis, is presumed to be established or reported [31]. The associated area includes 1,295 counties (N = 651 counties with established status, N = 644 counties with reported status). Results of univariable models (each socioeconomic variable individually), the final (reduced) multivariable model including multiple socioeconomic variables together, and the final (reduced) multivariable model including multiple socioeconomic variables and two ecological variables together are provided. Incidence was modeled using case counts (annual numbers of reported cases of human monocytic ehrlichiosis summed during 2007–2013 in each county); county population size in 2010 was included in the models as an offset term. Values for socioeconomic and ecological variables were centered by subtracting the mean and scaled by dividing each value by its centered standard deviation. Sources of all variables are provided in S1 Table. (PDF) [file pone.0204609.s009.pdf]

S7 Table. Results of county-level subset analyses using general linear mixed modeling to quantify associations between the incidence of human monocytic ehrlichiosis with six racial/ethnic and socioeconomic variables (socioeconomic variables) and two ecological variables. Subset analyses were limited to the subset of the 2,695 counties included in the full analyses in which *Amblyomma americanum,* the primary tick vector for human monocytic ehrlichiosis, is presumed to be established or reported [31].

	Univariable models			Multivariable model		Multivariable model with ecology	
**Variable**	**Coefficient, SE**	**P value**	**R^2 value of model**	**Coefficient, SE**	**P value**	**Coefficient, SE**	**P value**
Percent of housing units vacant	0.2728, 0.0492	<0.0001	0.5921	0.2195, 0.0481	<0.0001	0.1720, 0.0472	0.0003
Percent of population living below the poverty line	0.0733, 0.0585	0.2100	0.5854				
Percent of population classified as white non-Hispanic	0.3788, 0.0629	<0.0001	0.5988	0.2362, 0.0662	0.0004	0.1840, 0.0629	0.0034
Percent of population with a bachelors degree or higher	-0.2076, 0.0479	<0.0001	0.5904				
Percent of population unemployed	0.1096, 0.0673	0.1030	0.5865				
Per capita number of property crimes	-0.2770, 0.0485	<0.0001	0.5996	-0.1780, 0.0512	0.0005		
Density of white-tailed deer						0.2104, 0.0570	0.0002
Percent cover of forests						0.4331, 0.0587	<0.0001
**Conditional estimated R^2 value of model**				0.6083		0.6479	

(light grey)	Not included in model
(dark grey)	Included but not significant in final (reduced) model
